# Effects of Prolonged Antibiotic Therapy in Lung Abscesses—Analysis of Case Series

**DOI:** 10.1155/crpu/5976252

**Published:** 2025-10-06

**Authors:** Agata Anna Lewandowska, Dorota Waśniowska, Krzysztof Bronisz, Cezary Rybacki, Michał Graczyk, Helena Mirus-Arabik, Małgorzata Kołodziej, Aleksandra Gaczkowska, Ola Duszyńska

**Affiliations:** ^1^Clinical Department of Pulmonology, Allergology and Pulmonary Oncology, 10th Military Clinical Hospital with Polyclinic, Bydgoszcz, Poland; ^2^Clinical Department of Radiology, 10th Military Clinical Hospital with Polyclinic, Bydgoszcz, Poland; ^3^Faculty of Medicine, Bydgoszcz University of Science and Technology, Bydgoszcz, Poland; ^4^Department of Palliative Care, Collegium Medicum in Bydgoszcz, Nicolaus Copernicus University, Toruń, Poland; ^5^Clinical Department of Oncology, Oncology Center of Prof. Franciszek Lukaszczyk, Bydgoszcz, Poland

**Keywords:** antibiotic therapy, CT lesions, lung abscess, lung infection, necrotic, pneumonia, resistance

## Abstract

The incidence and mortality rate of lung abscess cases have declined significantly following the widespread introduction of broad-spectrum antibiotic therapy. Administration of antibiotics is considered the main treatment option, replacing invasive management, which currently is reserved only for selected patients. Four cases of patients with large lung abscesses analyzed in the article demonstrate the effectiveness of prolonged antibiotic therapy in the form of clinical improvement and regression of lesions imaged with computed tomography (CT) scans, in the absence of surgical drainage. However, the lack of a comparator group undergoing surgical interventions limits the ability to generalize the findings. The article highlights multiple diagnostic and management challenges clinicians face when treating complicated lung abscesses; however, the presented evidence is limited by a small sample size and lack of controls. Although the incidence of lung abscesses has dropped, they are still frequently seen in pulmonology, surgery, pediatrics, and internal medicine departments. In the face of the worldwide antimicrobial resistance crisis, the choice of effective antibiotic therapy remains a challenge, and there is no consensus on the duration of the treatment, as well as specific timing for introducing surgical intervention. As there are no high-quality recommendations or international studies evaluating the epidemiology of lung abscesses in the 21st century, further research seems necessary to help clinicians make appropriate therapeutic decisions.

**Summary**

• The incidence and mortality rate of lung abscess cases have declined significantly following the widespread introduction of broad-spectrum antibiotic therapy and advanced intensive care techniques.
• Despite the progress, diagnostic and treatment methods for both acute and chronic lung abscesses have received diminishing attention in 21st century medical literature.
• This does not change the fact that this disease, common in various age groups, is still frequently encountered and treated in pulmonology, surgery, pediatrics, and internal medicine departments.
• In treatment, clinical experience in the responsible use of long-term empirical and targeted antibiotic therapy, as well as a sense of timing in undertaking or withholding surgical intervention, is of primary importance.
• The findings presented in the article are observational and cannot be generalized without further comparative studies.
• Adding to the literature prospective clinical research and guidelines regarding current management in lung abscesses could help clinicians make appropriate therapeutic decisions.

## 1. Introduction

A lung abscess is a necrotic cavitary lesion of the lung parenchyma caused by a microbial infection of the lung [1–3]. In terms of etiology, lung abscesses can be divided into primary, if they occur in the absence of underlying pulmonary conditions in healthy individuals in all age groups, often aspiration-related, as well as secondary—in the presence of preexisting pulmonary lesions and an immunocompromised state [2–5]. They are also classified as acute (with a duration of less than 6 weeks) and chronic (more than 6 weeks) [2, 6]. In the literature from the 20th century, authors distinguished a subacute phase [7] with a duration of 6 weeks to 3 months [5]. Further classification is based on the pathogenic microorganism, such as anaerobic bacteria (e.g., *Peptostreptococcus*, *Bacteroides*, *Fusobacterium*, and *Prevotella*), pyogenic bacteria, fungi, and parasites [2, 8–11]. Among the causes of primary lung abscesses, excessive alcohol consumption is considered the most common predisposing factor in previously healthy patients [6, 12, 13], who frequently turn out to be infected with *Staphylococcus aureus*, *Klebsiella pneumoniae*, and *Streptococcus pyogenes* [1, 2, 14, 15]. Poor dental hygiene, though often overlooked during initial assessment, represents another significant risk factor for developing lung abscesses [2, 15].

The process of abscess formation often starts as lung inflammation, followed by necrosis, advancing fibrosis, all together resulting in the destruction of lung parenchyma and cavitation [16]. Lung abscesses can be indolent or symptomatic, in which case patients present a variety of ailments, such as fever, fatigue, loss of appetite, dyspnea, chest pain, and weight loss, as well as productive and nonproductive cough (depending on the contact of the abscess with the respiratory tract) [2, 17, 18]. If communication with the bronchus is present, drainage of the abscess occurs by productive cough, in some cases accompanied by hemoptysis [19] if erosion to the bronchial vessels occurs [20]. The clinical presentation and ultimate prognosis depend on multiple factors, including the specific pathogen involved, disease stage at diagnosis, immune system function, patient age, and presence of comorbidities [21, 22].

The diagnosis is based on x-rays, ultrasonography, and computed tomography (CT), which typically show a thick and irregular lung cavity with an air–fluid level [3, 16, 23, 24]. Differential diagnosis includes other types of possible infections, excavating tuberculosis, tumors, cystic lesions, and various malformations of the bronchial tree; however, the radiological sign of air–fluid level is seldom present in the above conditions [3, 19, 25], in which case CT provides the most detailed information [2, 26, 27].

Before the advent of antibiotics, acute lung abscesses carried an extremely high mortality rate, proving fatal in most cases [28]. The discovery of the open drainage operation in the early decades of the 20th century decreased the mortality rate; nevertheless, it was the advent of systemic antibiotics that showed the most satisfying outcome [19]. Early and common use of antimicrobials, together with advanced management of unconscious hospitalized patients, also contributed to the decrease in lung abscess cases in general [17]. Therefore, antibiotic therapy is considered the main treatment option, potentially accompanied by microbiological analysis of sputum or bronchial aspirate to assist in the ongoing treatment [2, 18]. Secondary lung abscesses, related to structural or functional lung disorders [16], show a much worse prognosis with a relatively high mortality rate, even as high as 75%, despite adequate antibiotic therapy [1]. If clinical improvement is achieved by conservative treatment, no surgical intervention should be required and, unlike empyema, is only reserved for failed antibiotic therapy lasting at least 6 weeks [19, 20, 26]. Invasive techniques, necessary in only about 10% of lung abscess patients [29], include percutaneous, endoscopic, or surgical drainage, as well as open surgical removal of lung lesions [30]—associated with high morbidity and mortality [31].

The case series enrolled patients with radiologically confirmed lung abscesses of at least 50 mm in size, treated conservatively between 2022 and 2024, who attended at least one follow-up visit after the treatment termination (in case of recovery) and consented to be included in the study. Several patients were excluded from the study due to incomplete inclusion criteria and follow-up loss, despite scheduled hospitalizations and contact attempts. In order to enhance reproducibility, an institutional protocol was established. Patients' eligibility for participation in the study was determined after retrospective analysis of medical history and chest CT images by a team of pulmonology and radiology specialists, with particular emphasis on the initial examination and the first follow-up after 5–8 weeks of oral antibiotic therapy. The presented cases demonstrate the efficacy of extended antibiotic therapy resulting in clinical improvement and significant regression of pulmonary lesions documented by CT imaging. Additionally, the article is aimed at highlighting the lack of high-quality recommendations by presenting multiple difficulties encountered by clinicians regarding the management of large and complicated lung abscesses.

## 2. Case 1

A 30-year-old patient was brought to the emergency department on the 1st of February 2024, suffering from increasing dyspnea for approximately 2 weeks, weakness, and elevated body temperature, together with a productive cough for about 5 days. The patient expectorated large amounts of purulent sputum. Oral antibiotic therapy based on clarithromycin was attempted at primary care due to a suspected respiratory tract infection 2 days prior to arrival at the hospital. The patient was not previously treated for chronic diseases or taking any medications on a regular basis. He initially denied excessive alcohol consumption, though later admitted to heavy drinking only a few days before presentation.

**Figure 1:**
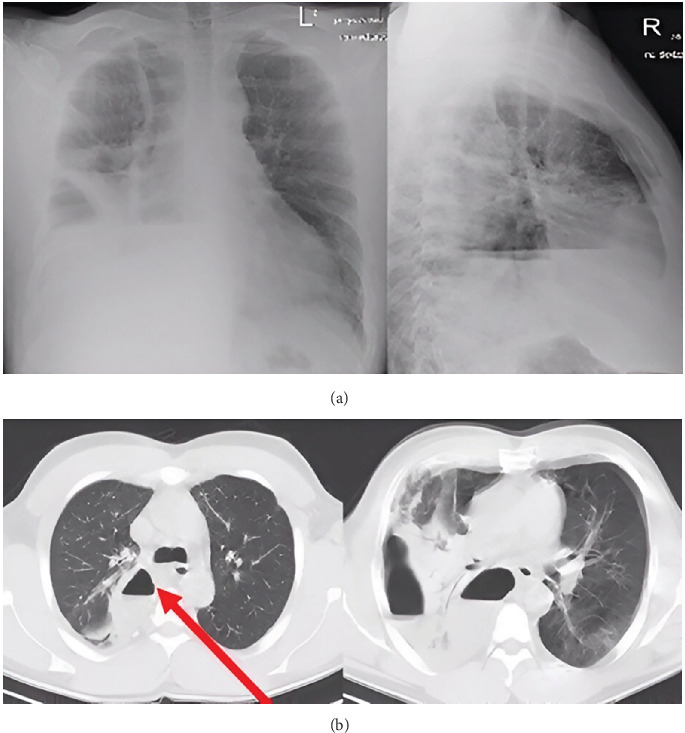
(a) Chest x-rays and (b) CT scans performed on the day of the patient's admission to the hospital (1st of February 2024)—Case 1. (b) The abscess wall is marked with an arrow.

During physical examination in the emergency department, the patient's condition was assessed as severe; he suffered from respiratory failure with capillary blood saturation at 70% without oxygen supplementation. On auscultation, the alveolar murmur was attenuated over the right lung, with bilateral crackles. In the laboratory tests performed, C-reactive protein (CRP) was 295 mg/L, procalcitonin (PCT) was 0.45 ng/mL, and white blood cell (WBC) was 42 G/L; mild hyponatremia was observed. The presence of fluid in the right pleural cavity with a fluid–gas level visible on the x-ray raised suspicion for a lung abscess (Figure 1a). An urgent CT scan of the chest with contrast was performed (Figure 1b).

CT (Figure 1b) confirmed the presence of two extensive gas spaces with fluid levels consistent with purulent lesions next to the spine and along the posterior–lateral chest wall, measuring 145 × 110 × 50 mm and 267 × 133 × 50 mm. Massive areas of inflammatory parenchymal thickening were visualized in both lungs.

Negative antigen test results for COVID-19, influenza, and respiratory syncytial virus were obtained. The patient was admitted to the pulmonology department with a diagnosis of the right lung abscess accompanied by bilateral pneumonia and suspected empyema.

In the department, empirical antibiotic therapy was started intravenously—piperacillin/tazobactam (4 g + 0.5 g) every 6 h, and electrolyte disturbances were leveled. A gradual decrease in inflammatory markers was observed—on the 12th day of the treatment, CRP was 41.5 mg/L, PCT was 0.21 ng/mL, and WBC was 16.30 G/L. In the sputum culture, a scanty growth of *Candida* species was observed, which was considered a contamination of the sample. No microorganisms were cultured in blood and urine samples.

**Figure 2:**
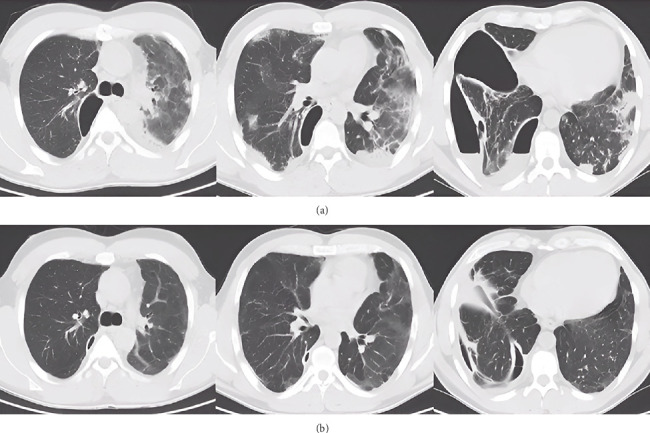
Follow-up CT scans of the patient's chest (Case 1) performed on the (a) 20th of February 2024 and (b) 27th of March 2024.

On the 13th day, due to the observed increase in CRP up to 65.2 mg/L, amikacin was added intravenously at a dose of 500 mg every 12 h. After the modification, the treatment was continued for another 9 days. A follow-up CT scan of the chest with contrast on the 20th of February (Figure 2a) showed a large amount of gas in the right pleural cavity with a level of fluid content (probably pyopneumothorax), together with a small cavity after a drained abscess with features of a bronchopleural fistula and a trace chamber with gas and fluid at the top of the left lung. The evolution of bilateral inflammatory lesions was evident, accompanied by reactive lymphadenopathy in the mediastinum.

Clinically, since the admission to the hospital, the patient presented a gradual improvement in general well-being, together with a progressive reduction in the need for oxygen supplementation, to a flow of 2 L/min through a nasal cannula. Due to the accompanying empyema, surgical treatment was considered. However, taking into account the significant clinical improvement and continuous active drainage, it was decided to continue the conservative treatment instead.

In accordance with the decision, the patient was discharged home due to his good general condition and a significant reduction in inflammatory markers. Oral antibiotic therapy was recommended after discharge: cefixime 400 mg every 24 h and moxifloxacin 400 mg every 24 h for the period of 5 weeks, until a follow-up hospitalization in the pulmonology department. Additionally, due to persistent features of hypoxemic respiratory failure, temporary oxygen therapy with a flow rate of 1–2 L/min was recommended at home.

After 1 month, the patient was readmitted to the pulmonology department for a posttreatment follow-up. The patient's general condition was assessed as good. On auscultation, there was a normal alveolar murmur with a quieting over the base of the right lung. Capillary blood saturation was 98% without oxygen supplementation. The patient discontinued oxygen therapy about 2 weeks after discharge due to capillary blood saturation measurements of 95%–98% and the absence of dyspnea. He affirmed that he had been taking antibiotic therapy as prescribed. The patient also mentioned deterioration of exercise tolerance, with gradual improvement since the discharge in February. In the laboratory tests performed that day, inflammatory markers were not elevated. CT imaging performed on the 27th of March 2024 revealed two longitudinal thick-walled cavities in the right lung without fluid content or interconnectivity, demonstrating significant regression of the previously identified lesions (Figure 2b). Radiologically, these appeared as fibroscar-like changes consistent with postabscess and postpneumonia resolution, additionally complicated with pleural empyema.

In the view of such significant regression of radiological lesions, nonelevated inflammatory markers, and the patient's good general condition, the decision was made to discontinue antibiotic therapy. Unfortunately, despite attempts to contact the patient, he did not return for another follow-up hospitalization.

## 3. Case 2

A 46-year-old patient was brought to the emergency department on the 12th of August 2022 because of dyspnea accompanied by productive cough, hemoptysis, and fever, present for 5 days. Due to suspected respiratory infection, azithromycin was started in primary care 2 days earlier. A few days before hospital admission, the patient had attended a large music festival where he consumed significant amounts of alcohol and recreational stimulants. He also had a history of pharmacologically compensated hypertension.

**Figure 3:**
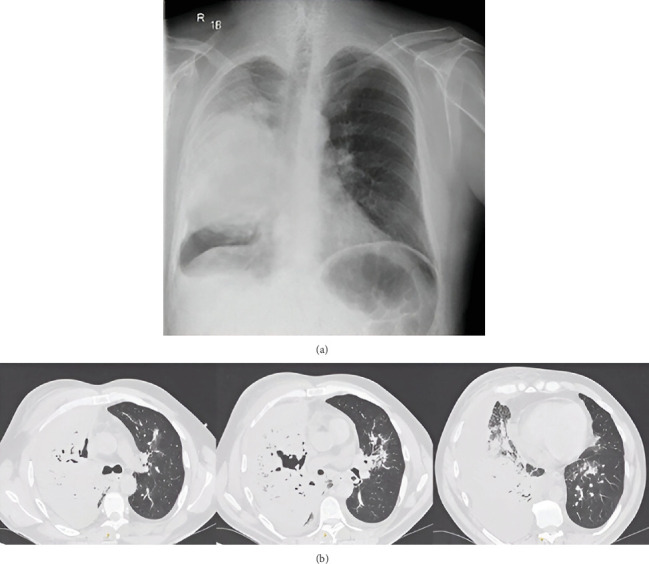
(a) Patient's chest x-ray performed on admission on the 13th of August 2022. (b) CT scans of the chest taken on the 17th of August 2022—Case 2.

On physical examination in the emergency department, the patient's condition was clinically stable. Chest auscultation revealed crackles on the right side together with a muted respiratory murmur at the base. In the performed laboratory tests, CRP was 477 mg/L, PCT was 6.84 ng/mL, and mild hypokalemia was present. Chest x-ray revealed complete shadowing of the middle and partially lower right lung field (Figure 3a).

The patient was admitted to the pulmonology department initially with a diagnosis of right-sided pneumonia and suspected sepsis. Empirical intravenous antibiotic therapy was started in the form of piperacillin/tazobactam at a dose of 4 g + 0.5 g every 6 h. A gradual decrease in inflammatory markers was observed—CRP dropped to 244 mg/L and PCT to 1.02 ng/mL. Blood, sputum, and urine cultures were drawn. HIV and tuberculosis testing turned negative. Blood and sputum cultures were positive for *Klebsiella pneumoniae.*

A CT scan (Figure 3b) of the chest showed an extensive abscess of the right lung with a multilocular cavitation involving the upper lobe of the lung, with fluid in both pleural cavities (up to 50 mm on the right side and a trace of fluid on the left side). On the left side, small peribronchial infiltrative foci were noted. In the mediastinum, several lymph nodes were up to 12 × 13 mm.

Due to an increase in inflammatory markers on the sixth day, intravenous amikacin at a dose of 500 mg every 12 h was added to the treatment according to the antibiogram. Gradual improvement in general condition was observed, together with the expectoration of a large amount of blood-colored sputum.

On the 12th day of hospitalization, antibiotic therapy was modified again due to a renewed rise in CRP. The previously administered antibiotics were discontinued. Ceftriaxone at a dose of 2 g every 24 h and ciprofloxacin at a dose of 400 mg every 12 h were started intravenously.

**Figure 4:**
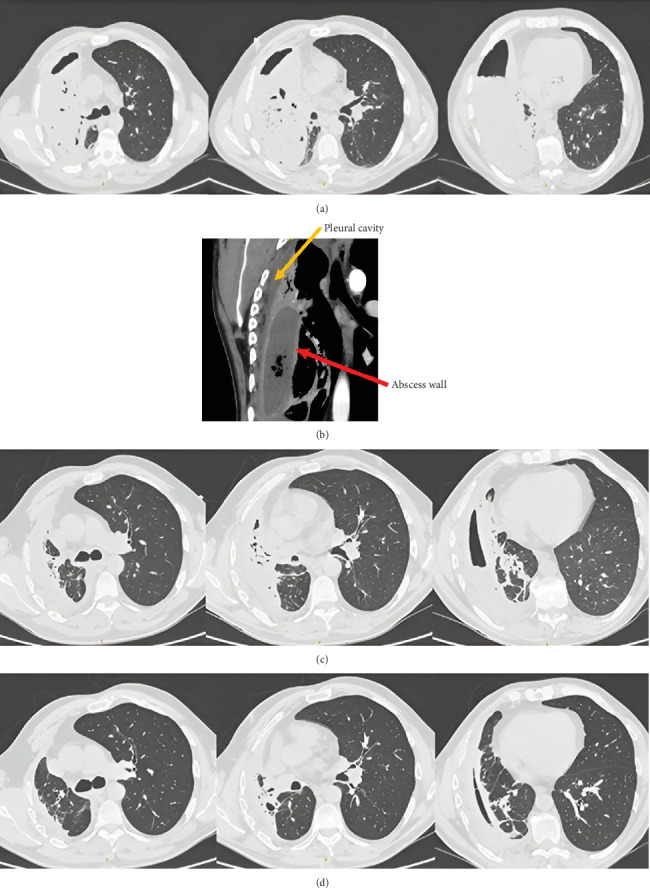
Follow-up CT scans performed on the (a) 1st of September 2022, (b) 6th of October 2022, (c) 25th of October 2022, and (d) 28th of December 2022—Case 2.

Bronchoscopy showed edema of the bronchial mucosa of the right lung and almost complete closure of the bronchi to the right superior lobe (B1 and B2). A follow-up CT scan (Figure 4a) of the chest revealed almost complete parenchymal disintegration of the upper and middle lobes of the right lung with the formation of an extensive multichambered fluid reservoir. The lower lobe on that side was airless, compressed by fluid. The left lung was within normal limits. Slightly enlarged, reactive lymph nodes were noted in the mediastinum.

Clinically, since the admission to the department, further improvement in the general condition and subsidence of hemoptysis was observed. Therefore, surgical treatment was withheld in the acute phase. After approximately a month of therapy, CRP was 68 mg/L and PCT was 0.08 ng/mL. At that time, the hospital treatment was terminated according to the patient's wishes. Oral antibiotic therapy was continued in the form of cefixime 400 mg every 24 h and ciprofloxacin 500 mg every 12 h for 5 weeks until a follow-up hospitalization in the pulmonology department.

However, 6 days after discharge, the patient returned to the emergency department with fever for 4 days, accompanied by a cough with expectoration of purulent sputum. In laboratory tests, CRP was 50 mg/L. Chest x-ray revealed no significant changes compared to the previous examinations.

The patient was readmitted to the pulmonology department, and the previous antibiotic therapy (ceftriaxone and ciprofloxacin) was continued intravenously. Despite this approach, the patient's condition deteriorated, as evidenced by increasing CRP levels reaching 121 mg/L, persistent fever, and continued production of purulent sputum. Intravenous imipenem/cilastatin at a dose of 0.5 g + 0.5g was started 4 days after readmission. Following this intervention, clinical improvement was observed. The fever gradually stopped and the amount of expectorated sputum decreased. A CT scan taken 2 weeks after admission (6th of October) showed a significant regression of the extensive abscess, measuring approximately 160 × 35 × 120 mm (Figure 4b). Approximately 3 weeks after admission, inflammatory markers increased again (CRP was 108 mg/L). Vancomycin at a dose of 2 g every 12 h intravenously was added to the applied treatment.

A CT scan of the chest taken on the 25th of October (Figure 4c) showed regression of the extensive fluid reservoir with air, after partial necrosis of the right upper lobe (third segment); the width of the residual cavity visible from the lateral side was about 25 mm. The second segment was airless with a preserved air bronchogram. Middle and lower lobes, as well as the first segment on that side, had reduced airiness. Traces of fluid were observed in the right pleural cavity. The left lung is partially distended and within normal limits. The mediastinum is displaced to the right side. There are reactive lymph nodes in the mediastinum under the main carina.

In laboratory testing on the day of the CT scan, CRP was 30 mg/L and PCT was 0.05 ng/mL. The patient was again discharged from the hospital with the recommendation to continue oral antibiotic therapy: clarithromycin 500 mg every 12 h, cefixime 400 mg every 24 h, and levofloxacin 500 mg every 12 h for the next 2 months. The decision was based on improvement in general condition, regression of radiological lesions, and reduction of inflammatory markers.

Two months later, the patient was readmitted to the pulmonology department for a follow-up hospitalization. The patient was in good general condition; however, he reported decreased exercise tolerance. Inflammatory markers were not elevated. The CT scan (Figure 4d) showed a longitudinal, fibrotic cavity in the basolateral part of the right lung (described in the previous examinations—now without fluid), and its size reduced to about 80 × 13 × 80 mm. Extensive postinflammatory fibrosis in the lower lobe remained unchanged, and less severe lesions were visible in the upper lobe (the airiness slightly improved), while the middle lobe was still almost completely airless. Massive pleural thickening on this side was visible (up to 19–20 mm). The left lung was substitutively distended, without thickening or focal changes. The mediastinum was displaced to the right side, with several moderately enlarged reactive lymph nodes under the main carina.

The patient was discharged home with a recommendation for another follow-up, and antibiotic therapy was terminated.

**Figure 5:**
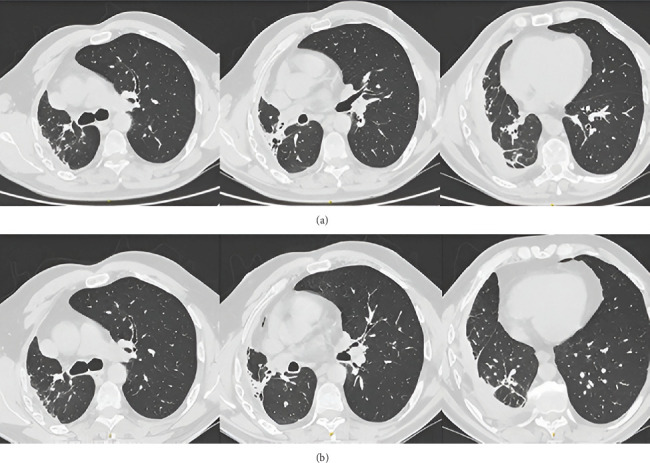
Follow-up CT scans performed on the (a) 7th of June 2023 and (b) 21st of February 2024—Case 2.

Next hospitalization took place 6 months later, in June 2023. On admission, the patient was in good general condition; he reported impaired exercise tolerance and pain in the right mediastinal region after exercise. In laboratory tests, inflammatory markers remained negative. The CT scan (Figure 5a) showed an airy, fluid-free cavity of 40 × 7 × 50 mm, located laterally in the right lung, limited by massive adhesions. Further marked slight improvement in the airflow of the right lung, with partial cirrhosis of the upper lobe and fibroaneurysmal changes in the lower lobe. The mediastinum was still displaced to the right side, and the left lung substitutively distended. There was massive thickening of the fibrotic pleura from the posterior lateral side on the right side (up to 18 mm). Pulmonary function testing revealed a severe restrictive ventilation disorder, along with severely reduced carbon monoxide diffusing capacity (DLCO).

The last follow-up took place 8 months later, in February 2024. On admission, the patient's general condition was good. He reported persistent deterioration of exercise tolerance, with no change since the last hospitalization, and experiencing pain in the right mediastinal region after exercise. The CT scan (Figure 5b) showed a residual, air-filled cavity after a drained right lung abscess measuring 19 × 5 × 40 mm. Other than that, the image of pulmonary parenchyma and mediastinal structures remained stable. The respiratory mechanics remained unchanged.

## 4. Case 3

**Figure 6:**
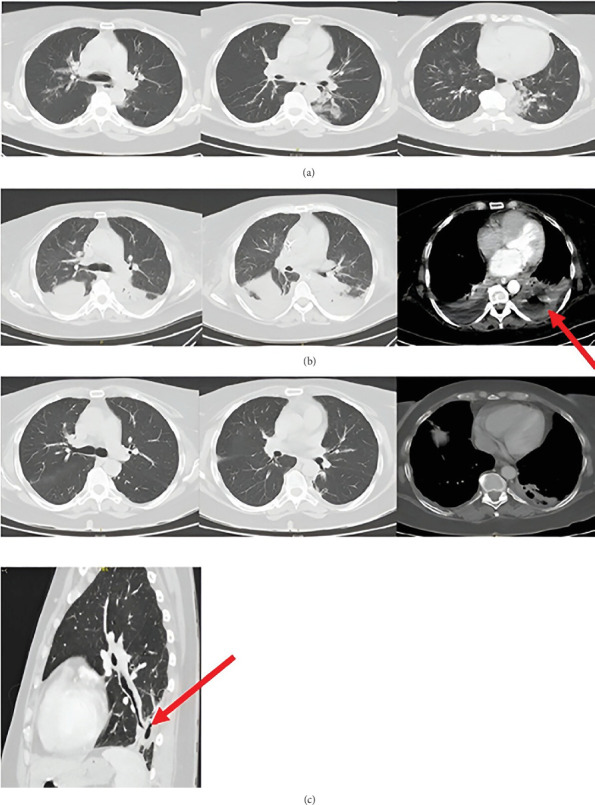
(a) CT scans performed on admission on the 14th of March 2024. Follow-up CT scans performed on the (b) 5th of April 2024 and (c) 29th of May 2024—Case 3. (b, c) The abscess is marked with an arrow.

A 53-year-old patient was brought to the emergency department on the 14th of March 2024 suffering from severe dyspnea, cough, and fever for several days. The medical history included cardiac arrhythmia and obesity (BMI 34.6 kg/m^2^). The patient's condition was assessed as severe. Chest auscultation revealed bilateral crackles over the lung fields. Oxygen therapy was required. Tests for influenza and COVID-19 were negative. In the laboratory testing, CRP was 99 mg/L and PCT was 2.28 ng/mL. Due to the clinical presentation suggesting possible pulmonary embolism, a chest CT with contrast was urgently performed, which excluded thromboembolic disease but revealed bilateral inflammatory parenchymal thickening with ground-glass opacities in the left lung (Figure 6a). The patient was admitted to the pulmonology department with the diagnosis of bilateral pneumonia.

Intravenous antibiotic therapy was started in the form of piperacillin/tazobactam 4.5 g every 6 h with amikacin 500 mg every 12 h due to poor general condition. No microbial growth was obtained in blood and urine cultures. Despite the treatment, the patient's condition progressively deteriorated; an increase in inflammatory markers (CRP > 200 mg/L and PCT > 5 ng/mL), impaired consciousness, and seizures were observed. Imipenem/cilastatin 500 mg + 500 mg every 6 h was started on the 6th day; piperacillin/tazobactam was discontinued. Cerebral edema and neuroinfection were excluded after a head CT scan, ophthalmologic examination, and cerebrospinal fluid analysis.

Due to the development of hypercapnic respiratory failure, noninvasive ventilation was introduced. Progressive alteration of mental status, fever, and respiratory failure was observed. After anesthesiological consultation, the patient was intubated and placed on mechanical ventilation 6 days after admission to the hospital. During bronchoscopy, a large amount of blood-stained mucus was aspirated from the bronchi of both lungs. The patient was transferred to the intensive care unit (ICU) for further treatment, where advanced mechanical ventilation techniques and analgosedation were implemented. Two days later, a follow-up CT scan of the chest showed an obstructed left lower lobe bronchus with massive atelectasis–inflammatory lesions on that side. Extensive inflammatory lesions, especially in the middle lobe, were visualized in the right lung.

Antibiotic therapy was modified according to the microbiological findings and antibiogram. Initially, cloxacillin with colistin was introduced with satisfactory results 5 days after admission to the ICU due to the growth of *Staphylococcus aureus* in three bronchial aspirate cultures, as well as concomitant *Staphylococcus hominis* septicemia. After about a week, inflammatory markers increased again—CRP was 203 mg/L and PCT was 9.22 ng/mL. During bronchoscopy, the left lower lobe bronchus was filled with thick purulent secretion. Due to the newly detected growth of *Acinetobacter baumannii* in both blood cultures and bronchial aspirate, the antibiotic therapy was again modified to imipenem/cilastatin and colistin after analysis of subsequent antibiograms. A gradual decrease in inflammatory markers was observed.

After the patient's gradual improvement, it was possible to terminate the ventilator therapy and the infusion of catecholamines. Imipenem/cilastatin was discontinued. A follow-up CT scan of the chest performed 2 weeks after admission to the ICU (Figure 6b) showed almost complete shadowing of the left lower lobe with an isolated fluid reservoir with the presence of gas, measuring approximately 49 × 32 × 62 mm—an abscess. There was fluid present bilaterally in the pleural cavities and in the right interlobar fissure.

The patient was transferred to the pulmonology department with the diagnosis of the left lung abscess a day later (6th of April). Shortly after admission, inflammatory markers increased again—CRP was 100 mg/L and PCT was 0.32 ng/mL. Intravenous ceftriaxone 2 g every 24 h and levofloxacin 500 mg every 12 h were added to the previously received colistin at a dose of 3 million IU every 8 h. A gradual normalization of CRP and PCT was achieved. The patient was discharged from the department after 10 days of treatment in good general condition, with no need for oxygen therapy. CT scan was not repeated due to the significant clinical improvement. It was recommended to take oral antibiotic therapy for the next 5 weeks in the form of cefixime 400 mg every 24 h and levofloxacin 500 mg every 12 h.

The patient was readmitted to the pulmonology department for a follow-up hospitalization after a month and a half, on the 28th of May 2024. The condition was assessed as good. The patient reported an occasional cough of low intensity. Laboratory testing showed no clinically significant abnormalities—CRP and PCT were normal. A CT scan revealed only a small residual abscess cavity in the 10th segment of the left lung surrounded by fibrotic lesions, indicating substantial resolution of the previously identified lesions (Figure 6c). It was decided to discontinue antibiotic therapy and further observation.

## 5. Case 4

**Figure 7:**
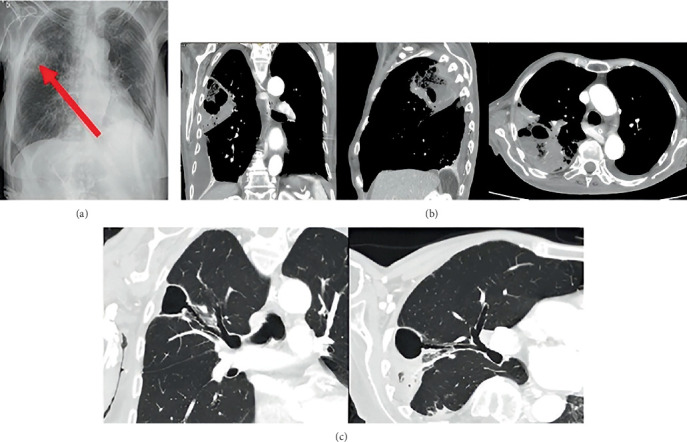
(a) A chest x-ray performed on admission on the 4th of October 2022. CT scans performed on the (b) 7th of October 2022 and (c) 22nd of November 2022—Case 4.

An 85-year-old patient was brought to the emergency department on the 4th of October 2022 due to fever and marked reduction in oral intake. The patient suffered from end-stage Alzheimer's disease with profound dementia, was chronically bedridden, severely malnourished, and unable to communicate verbally. The patient did not require oxygen therapy; auscultation did not reveal any abnormalities. In laboratory testing, elevated inflammatory markers were observed (CRP was 195 mg/L and PCT was 0.77 ng/mL), as well as anemia with HGB 92 g/L and a urinary tract infection. A chest x-ray showed a translucent, irregularly shaped cavity in the right lung, measuring approximately 38 mm—possibly an abscess (Figure 7a).

The patient was admitted to the pulmonology department due to a suspected lung abscess and a concomitant urinary tract infection. Blood and urine cultures were obtained. Empirical intravenous antibiotic therapy was started in the form of ciprofloxacin 400 mg every 12 h and ceftriaxone 2 g every 24 h. A CT scan of the chest performed on the third day (Figure 7b) showed diffuse infiltrative lesions accompanied by an abscess in the process of evolution located in the upper lobe of the right lung, measuring approximately 50 mm. In addition, inflammatory lesions were visible in the sixth segment on this side. On the left side, a trace of fluid was present in the pleural cavity, together with segmental airlessness of the dorsal part of the lower lobe and preserved air bronchogram.

The diagnosis of a lung abscess was confirmed. Sputum was analyzed for mycobacteria and nonspecific flora; however, no pathogenic microorganisms were cultured. The urine culture was positive for *Escherichia coli*. During hospitalization, the patient required continued feeding by a gastric probe and still did not make verbal contact due to advanced dementia. A gradual decrease in inflammatory markers was observed to CRP 23.8 mg/L and PCT 0.07 ng/mL. After 9 days of treatment and satisfactory clinical improvement, it was decided to discharge the patient with the recommendation of oral antibiotic therapy for the next 5 weeks in the form of cefixime 400 mg every 24 h and ciprofloxacin 500 mg every 12 h.

The patient was readmitted to the pulmonology department for a follow-up hospitalization approximately 5 weeks later, in November 2022. Inflammatory markers remained low. A CT scan (Figure 7c) showed a significant regression of the right lung lesions. A residual abscess cavity measuring 30 mm, with a trace of fluid, was visualized in the upper lobe on that side. Partial airlessness of the basal part of the left lower lobe was assessed as secondary to the mucus retention in the lower lobe bronchus.

Due to the visualized atelectasis of the left lower lobe, bronchoscopy was performed. A moderate amount of mucus was aspirated from the bronchial tree. Microbiological culture of the bronchial aspirate was negative. Despite the regression of radiological lesions, the patient's general condition did not change, probably due to advanced age, comorbidities, and dementia. Before the planned discharge, cardiac arrest occurred, and the patient was pronounced dead.

## 6. Discussion

Primary abscesses are often related to the aspiration of oral material, poor dental hygiene, alcoholism, drug usage, and poor consciousness [1, 24, 32] (Patients 1 and 2). Both Patients 1 and 2 actively expectorated large amounts of sputum, indicating that the drainage of the abscesses occurred due to the communication with bronchi. Usually, no major diagnostic difficulties are encountered in the case of well-confined primary lung abscesses, especially in the era of widely available CT. However, the diagnosis of extensive abscesses with concurrent widespread pulmonary inflammation poses considerable challenges for both pulmonologists and radiologists. The radiological image changes over time, and some patients may develop secondary complications, such as purulent pleural effusion or bronchopleural fistula (Patient 1). In the case of Patient 3, the lung abscess was a secondary complication in the course of severe bilateral pneumonia. Key radiological findings for each patient are presented in Table 1.

The main treatment of primary lung abscess consists of the administration of broad-spectrum antibiotics (empiric coverage) with a mortality rate of less than 10% [1, 14, 33]. The failure of conservative treatment can be a result of the virulence of the pathogen, inadequate concentration of the drug in the lesion, and the presence of underlying lung disorders and reduced lung compliance [31]. The penetration and efficacy of antibiotics are limited and depend on the degree of abscess maturation, pH, protein binding, and activity of bacterial enzymes [34]. The drug penetration from plasma into the abscess fluid is majorly obstructed by several morphological and anatomical barriers, including factors like the thickness of the collagen wall, the degree of vascular necrosis around the cavity, the viscosity of fluids inside the capsule, and the coefficient of diffusion [35, 36]. The permeability of the abscess wall is significantly influenced by infection duration, encapsulation advancement, and the extent of surrounding fibrosis [35, 37]. Due to the generally poor drug concentration at the abscess site and a substantial delay in reaching the onset of antimicrobial effect, the treatment duration must be extended accordingly [1, 34]. Several adjunctive therapies are being explored to potentially enhance the effectiveness of antibiotics, such as physiotherapy, mucolytics, or targeted drug delivery [38–40].

**Figure 8:**
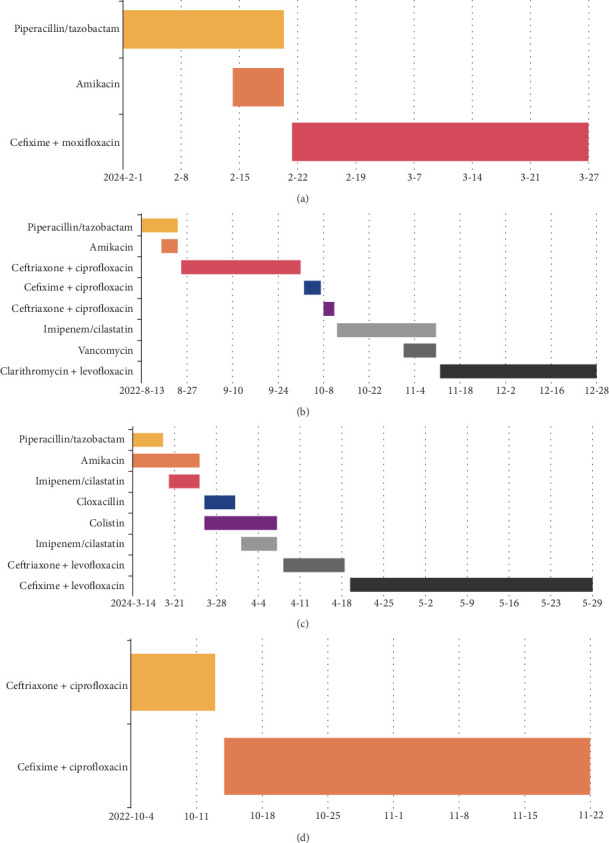
Treatment duration in Patients (a) 1, (b) 2, (c) 3, and (d) 4.

All of the above factors could have contributed to the need for prolonged pharmacotherapy and multiple drug modifications, especially in the case of Patient 2. Unfortunately, despite the final effectiveness of the treatment, clinical improvement may not always be achievable, particularly in elderly patients with multimorbidity (Patient 4). However, the lung abscess cannot be considered the cause of the patient's death, which occurred after the completion of antibiotic therapy and documented regression of the lesions. A summary of the reviewed patients and the courses of their treatment is presented in Table 2 and Figure 8.

The choice of effective antibiotic therapy remains a challenge, especially in the face of global antibacterial drug resistance [41]. Antibiotic adjuvants, such as beta-lactamase inhibitors, reduce bacterial resistance mechanisms and therefore increase the antibiotic's efficacy to successfully treat both Gram-positive and Gram-negative pathogens [42]. However, anaerobic bacteria resistance has become increasingly unpredictable, even to the most active drugs [43]. Although penicillin was historically the first-line treatment for primary lung abscesses, it has been supplanted by beta-lactam/beta-lactamase inhibitors, clindamycin, fluoroquinolones, metronidazole, and vancomycin due to resistance in approximately 15%–20% of anaerobic bacteria [6, 20, 44–47]. Antimicrobial activity of several commonly used antibiotics is presented in Table 3 [43].

The empiric treatment should target pathogens such as *Streptococcus*, *Staphylococcus* spp. if hematological dissemination is suspected, and *Pseudomonas* spp.—in patients with elevated risk of aspiration [28]. Amoxicillin and clavulanic acid are suggested in the first scenario, with vancomycin if methicillin-resistant *Staphylococcus aureus* (MRSA) coverage is necessary [28]. In the other case, piperacillin/tazobactam is recommended [28]. MRSA infection warrants particular consideration in nosocomial settings or when initial antibiotic therapy proves ineffective [48]. However, rational limitation of unnecessary anti-MRSA therapy by an early collection of sputum and blood cultures seems crucial in the face of growing antibiotic resistance, especially since anti-MRSA coverage can often be added or discontinued without necessarily adjusting the ongoing Gram-negative antibiotic therapy [49]. In the presented cases, the most commonly used initial empiric antibiotic regimen included a beta-lactam with a beta-lactamase inhibitor, with further modifications dictated by the clinical context and culture results. In Patients 1, 2, and 3, piperacillin/tazobactam was the first antibiotic included in the treatment. In the case of Patients 2 and 3, the choice was additionally dictated by the suspicion of concurrent sepsis and poor general condition. The administration of amikacin shortly after admission in Patient 3 was justified by the patient's acute deterioration accompanied by suspected neuroinfection. In Patient 4, empiric coverage was also targeted at the concomitant urinary tract infection.

As various pathogens present similar symptoms in lung abscesses, it is important to draw blood and sputum/bronchial aspirate cultures to attempt to identify them and optimize antibiotic therapy [50] if a microorganism with antibiotic resistance is isolated [21]. Such management was implemented in Patients 2 and 3; however, even targeted antibiotic therapy based on the antibiogram of blood and sputum cultures did not guarantee a satisfactory outcome, and further changes in the treatment were required. In some instances, diagnostic culture results remain unobtainable, forcing clinicians to rely solely on empirical antibiotic therapy, as observed in Patients 1 and 4.

Negative or contaminated cultures represent a major source of clinicians' frustration as they often result in diagnostic uncertainty and inadequate treatment strategies [51]. Collecting sputum using a noninvasive method requires certain preparations in order to avoid difficult-to-interpret samples contaminated with oral secretions [52]. The procedure must be conducted in a sanitary regimen after providing the patient with instructions regarding the procedure [52]. Ideally, samples should be collected prior to antibiotic administration and promptly delivered to the laboratory to maximize diagnostic yield [52]. Invasive methods, such as bronchial aspirate collection during bronchoscopy, may provide valuable samples directly from the site of infection, therefore increasing the chance of identifying the pathogenic microorganism [53].

There is no consensus on the duration of the treatment as it is based on the clinical and radiological improvement [28, 54, 55]. To prevent therapeutic failure, treatment duration should extend no less than 6 weeks, and in certain cases, it may require 14–16 weeks [20, 28]. The duration of implemented antibiotic therapy and clinical recovery varied in the presented patients; however, in no case was it shorter than 6 weeks. In elderly patients, conducting chest physiotherapy followed by pulmonary rehabilitation after discharge is highlighted as an important factor in supporting recovery and improving respiratory function [21]. In the case of Patients 1 and 2, the deterioration of exercise tolerance was observed after the treatment termination. In Patient 2, the impairment in pulmonary function testing was still observed many months after the treatment, suggesting permanent loss of lung function due to the presence of postinflammatory lesions. Unfortunately, performing such follow-up was not possible in the case of Patient 1, who declined further observation. Patient 3 was additionally recovering after treatment in the ICU; however, during the last follow-up, the patient did not complain of impaired physical performance, and therefore pulmonary function testing was not performed. However, performing follow-up with spirometry could help assess long-term sequelae and the quality of life in patients treated for lung abscesses. Additionally, the lack of standardized outcome tools, such as self-report questionnaires assessing health-related quality of life (e.g., SF-36), may limit the generalizability of outcome comparisons.

In case of massive lesions and unsuccessful conservative treatment lasting for more than 6 weeks (chronic lung abscesses), pulmonary drainage is indicated to support the recovery process [8, 19, 56]. However, surgical management is associated with the risk of complications in the form of pleural empyema, bronchopleural fistula, or procedure-related bleeding [19] and is therefore currently required only in selected patients [6, 30, 57]. Conservative management yielded positive results in the presented cases of large lung abscesses, with none requiring surgical intervention at any stage of treatment. However, the outcomes were assessed majorly through radiological imaging and inflammatory markers, as pulmonary function testing or validated quality-of-life assessments were applied inconsistently. The generalizability of these observational findings is also confined by small sample size and the lack of follow-up uniformity among the patients, which has been enforced by the premature loss of contact or posttreatment clinical outcome. Another limitation is the lack of a comparator group that would undergo a surgical intervention.

The presented cases prove that the course of the treatment can be arduous and complicated, sometimes requiring many changes in the implemented pharmacotherapy. The initial antibiotic selection and differential diagnosis are further complicated and protracted by pending microbiological and radiological results, potentially leading to suboptimal therapeutic choices. It is worth mentioning that empiric coverage depends not only on differential diagnosis but also on the patient's characteristics, concomitant diseases, local microbial susceptibility, availability of antibiotics, potential drug intolerances, and toxicity [58].

A worldwide demand for novel antimicrobials displaying innovative modes of action is especially highlighted in the fields of natural product–derived and synthetic small molecules [59]. In addition to the continuous search for new antibiotic agents, clinical judgment in the appropriate use of the available ones should be a crucial part of the strategy [60]. Most lung abscesses respond to conservative management. However, progressive deterioration of clinical symptoms and the lack of radiological improvement despite prolonged intravenous antibiotic therapy generally warrant surgical intervention. The diversity of case reports in the literature underscores the complexity of problems encountered by clinicians when treating lung abscesses, thus emphasizing the need for additional high-quality clinical trials and evidence-based recommendations [61]. These could facilitate and standardize treatment management, both conservative and invasive.

## Figures and Tables

**Table 1:** Summarized radiological findings in Patients 1–4 diagnosed and treated for lung abscesses.

	**Patient 1**	**Patient 2**	**Patient 3**	**Patient 4**
Diagnosis date	1st of February 2024	17th of August 2022	5th of April 2024	7th of October 2022
Abscess size at diagnosis	145 × 110 × 50 mm	N/A	49 × 32 × 62 mm	50 mm
Date of treatment completion	27th of March 2024	28th of December 2022	29th of May 2024	22nd of November 2022
Abscess size at the end of treatment	N/A	80 × 13 × 80 mm	N/A	30 mm
Abscess location	Right lung, along the spine	Upper and middle lobes of the right lung	Lower lobe of the left lung	Upper lobe of the right lung
Presence of fluid	✓	✓	✓	✓
Presence of air	✓	✓	✓	
Empyema	✓			
Bronchopleural fistula	✓			
Fibrosis	✓	✓	✓	

**Table 2:** A summary of the reviewed cases.

	**Patient 1**	**Patient 2**	**Patient 3**	**Patient 4**
Age	30 years old	46 years old	53 years old	85 years old
Comorbidities	None	Hypertension	Cardiac arrhythmia and obesity	Alzheimer's disease, cachexia, anemia, and concomitant urinary tract infection
Causative organism	Unknown (negative sputum, blood, and urine cultures)	*Klebsiella pneumoniae* (blood and sputum cultures)	• *Staphylococcus aureus* (bronchial aspirate cultures) • *Staphylococcus hominis* (blood cultures) • *Acinetobacter baummanii* (blood and bronchial aspirate cultures)	• Unknown (negative sputum and blood cultures) • *Escherichia coli* (urine culture)
Antibiotic course	• Piperacillin/tazobactam (from the 1st–22nd day) • Amikacin (from the 13th–22nd day) • Oral cefixime and moxifloxacin (5 weeks after discharge)	• Piperacillin/tazobactam (from the 1st–11th day) • Amikacin (from the 6th–11th day) • Ceftriaxone and ciprofloxacin (from the 12th–36th day) • Oral cefixime and ciprofloxacin (from the 37th–43rd day) • Ceftriaxone and ciprofloxacin (from the 44th–48th day) • Imipenem/cilastatin (from the 49th–79th day) • Vancomycin (from the 69th–79th day) • Oral clarithromycin, cefixime, and levofloxacin (8 weeks after discharge)	• Piperacillin/tazobactam (from the 1st–5th day) • Amikacin (from the 1st–11th day) • Imipenem/cilastatin (from the 6th–11th day) • Cloxacillin (from the 12th–18th day) • Colistin (from the 12th–24th day) • Imipenem/cilastatin (from the 19th–24th day) • Ceftriaxone and levofloxacin (from the 25th–35th day) • Oral cefixime and levofloxacin (5 weeks after discharge)	• Ceftriaxone and ciprofloxacin (from the 1st–9th day) • Cefixime and ciprofloxacin (5 weeks after discharge)
Treatment duration	8 weeks	19 weeks	10 weeks	6 weeks
Complications	Bronchopleural fistula and pleural empyema	Residual extensive postinflammatory fibrosis and massive pleural thickening	Respiratory failure requiring ventilator therapy and ICU treatment and septicemia	Atelectasis of the left lower lobe and clinical deterioration
Outcome	Recovered with subsequent deterioration of exercise tolerance after 8 weeks of treatment	Recovered with permanent impairment of exercise tolerance and lung function	Recovered with no complaints of impaired physical performance	Deceased

Abbreviation: ICU, intensive care unit.

**Table 3:** Antimicrobial activity of several antibiotics commonly used in clinical practice [43].

	**Beta-lactamase-producing anaerobic Gram-negative bacilli**	**Other anaerobes**	**Gram-positive cocci**	**Enterobacteriaceae**
Penicillin		✓	✓	
Metronidazole	✓	✓		
Clindamycin	✓	✓	✓	
Amoxicillin/clavulanate Carbapenems Cefoxitin Piperacillin/tazobactam Moxifloxacin	✓	✓	✓	✓

## Data Availability

Data sharing is not applicable to this article as no datasets were generated or analyzed during the current study.
